# Beyond the crisis: building back better mental health care in 10 emergency-affected areas using a longer-term perspective

**DOI:** 10.1186/s13033-015-0007-9

**Published:** 2015-03-12

**Authors:** JoAnne E Epping-Jordan, Mark van Ommeren, Hazem Nayef Ashour, Albert Maramis, Anita Marini, Andrew Mohanraj, Aqila Noori, Humayun Rizwan, Khalid Saeed, Derrick Silove, T Suveendran, Liliana Urbina, Peter Ventevogel, Shekhar Saxena

**Affiliations:** Consultant to the World Health Organization (WHO), Seattle, USA; World Health Organization (WHO), Geneva, Switzerland; Ministry of Health, Palestinian Authority, Ramallah, West Bank occupied Palestinian territory; Section on Community Psychiatry, the Indonesian Psychiatric Association, Jakarta, Indonesia; Citizenship INGO, Rimini, Italy; CBM, Kuala Lumpur, Malaysia; WHO Country Office, Baghdad, Iraq; WHO Somalia Liaison Office, Nairobi, Kenya; WHO Regional Office for the Eastern Mediterranean, Cairo, Egypt; University of New South Wales, Sydney, Australia; WHO Country Office, Colombo, Sri Lanka; Municipalidad de la Ciudad de San Luis, San Luis, Argentina; United Nations High Commissioner for Refugees (UNHCR), Geneva, Switzerland

**Keywords:** Developing countries, Disasters, Health care reform, Health policy, Mental health, Mental health services, Refugees, War, (Source: MeSH)

## Abstract

**Background:**

Major gaps remain – especially in low- and middle-income countries – in the realization of comprehensive, community-based mental health care. One potentially important yet overlooked opportunity for accelerating mental health reform lies within emergency situations, such as armed conflicts or natural disasters. Despite their adverse impacts on affected populations’ mental health and well being, emergencies also draw attention and resources to these issues and provide openings for mental health service development.

**Case description:**

Cases were considered if they represented a low- or middle-income country or territory affected by an emergency, were initiated between 2000 and 2010, succeeded in making changes to the mental health system, and were able to be documented by an expert involved directly with the case. Based on these criteria, 10 case examples from diverse emergency-affected settings were included: Afghanistan, Burundi, Indonesia (Aceh Province), Iraq, Jordan, Kosovo, occupied Palestinian territory, Somalia, Sri Lanka, and Timor-Leste.

**Discussion and evaluation:**

These cases demonstrate generally that emergency contexts can be tapped to make substantial and sustainable improvements in mental health systems. From these experiences, 10 common lessons learnt were identified on how to make this happen. These lessons include the importance of adopting a longer-term perspective for mental health reform from the outset, and focusing on system-wide reform that addresses both new-onset and pre-existing mental disorders.

**Conclusions:**

Global progress in mental health care would happen more quickly if, in every crisis, strategic efforts were made to convert short-term interest in mental health problems into momentum for mental health reform.

## Background

Important progress in mental health service development has been made in selected countries [1,2] of the world. Yet, despite increasing international focus and action, major treatment gaps remain worldwide; in low- and middle-income countries [3,4], around 80% of people in need do not receive appropriate services [5]. Meanwhile, mental disorders remain among the most prevalent, burdensome, and undertreated health conditions globally. Recent estimates indicate that mental disorders are responsible for 22.7% of all years lived with disability [6].

A distinct but overlapping public health domain concerns mental health and psychosocial support in emergency settings. Numerous natural disasters, armed conflicts, and other major hazards occur around the world annually, many of which are followed by an influx of international aid agencies offering various types of psychosocial services and financial resources. Although the quality and effectiveness of these mental health services can be highly variable [7], there is no doubt that mental health and psychosocial support is important during and after emergencies [8]. This is because rates of diverse mental health problems increase as the result of emergencies [9] and at the same time, those with pre-existing mental disorders are rendered more vulnerable [10]. In addition, existing public health infrastructure - including that for mental health - is often weakened.

Until recently, the potential synergies between these two public health domains have been largely overlooked. This is understandable because humanitarian relief is usually short-term in nature, whereas mental health policy and service development is a process that usually involves years if not decades to reach fruition. Most actors involved in humanitarian emergencies focus on short-term mental health needs while giving little consideration of the mental health systems that will be left behind after their aid agencies depart.

These challenges notwithstanding, emergency situations are ripe opportunities to build sustainable mental health services for all people in need [11]. This is due to several factors. First, the presence of aid agencies combined with a surge of financial resources into affected areas present possibilities for development that might not have been realistic previously. Second, the destruction or degradation of health services as a result of emergencies - while presenting near-term challenges - also opens opportunities to build more efficient, person-centred systems of care. Third, media attention combined with well-placed empathy from the general public frequently makes mental health a political priority after an emergency. Within this context, decision-makers often become willing to allocate resources towards mental health and to consider options beyond the status quo. This is important given that lack of political will has been identified as the principal barrier to mental health services’ expansion in low- and middle-income countries [4].

Examples have been published [12-19] in which countries or territories have been able to build better mental health systems following emergencies, and some have identified common threads among some of these experiences [20,21]. Yet more information and analysis are needed to develop our understanding of how it is possible to build a better-quality mental health system after an emergency.

This article reinforces and extends current knowledge in the area. It is co-authored by people who were involved directly with the cases profiled and as such, who have detailed knowledge of what worked in their unique contexts. And importantly, the article reports not only major achievements, but also how challenges were overcome. Key lessons learnt emerging from these experiences are summarized.

## Case description

Ten diverse emergency-affected areas are the focus of this article. Highly-detailed descriptions of each case are beyond the scope of this article but have been published previously as part of a WHO report [22]. Cases were considered if they: a) represented a low- or middle-income country or territory; b) were initiated between 2000 and 2010; c) succeeded in making sustained changes in the mental health system to improve access to basic mental health services; and d) were able to be documented by experts involved directly with the case.

The situational contexts of the cases varied. Some areas endured or are still enduring prolonged armed conflict and political instability. Others experienced lengthy civil conflicts before being struck by natural disaster. One country experienced a rapid and large influx of displaced people from a neighbouring country. Cases differed further in their pre-existing mental health systems, and in the capacity of the governments to respond to the emergency. These distinctions are described in greater detail below.

### Afghanistan

Because of ongoing violence, by 2001 much of the qualified workforce and technical expertise had left Afghanistan. The country had just two qualified psychiatrists (neither of them working in mental health service delivery) and around 138 other health-care staff (general doctors and nurses) providing mental health services. There was no regular budget allocation for mental health services.

Steady progress was made after the fall of the Taliban government in 2001. Initially, health service delivery – including mental health care – was contracted to NGOs [12], while the government concentrated on regulation and policy-making. During this period, mental health was declared a priority area but funding was not allocated commensurately. This changed after 2004, when a new Minister of Public Health declared publicly that mental health issues were among his most urgent priorities. In 2005, the Ministry of Public Health re-established a department for mental health and drug demand reduction; this department benefited from the long-term secondment by the European Community of an expatriate mental health advisor. In 2008, a technical working group of the Ministry, WHO, and NGOs produced a full range of mental health training manuals for different health worker cadres (physicians, nurses, midwives, and community health workers). Furthermore, a new category of health worker was introduced: the psychosocial counsellor. These paraprofessionals participated in a 3-month classroom training followed by a 9-month supervised practice in a health facility as part of their education. In 2010, the government endorsed a new 5-year National Mental Health Strategy. Mental health has been included in the country’s Basic Package of Health Services and Essential Package of Hospital Services, and headway has been made in various provinces. For example, close to 100 000 people with mental health conditions were seen within Nangarhar Province’s primary health services from 2002 to 2011.

Financial sustainability of mental health services is an ongoing challenge. Afghanistan’s health sector is heavily dependent on international donors and, as such, is subject to varying priorities and timelines. Future levels of international health funding are unclear. Even if funds continue at current levels, health workers are often overburdened and have limited time to address the mental health needs of their patients. However, the government remains strongly committed to the integration of mental health services into primary health care and actively encourages donors to invest in mental health service delivery by paying for the retraining of all health staff and creating new positions for psychosocial counsellors.

### Burundi

Modern mental health services were almost non-existent prior to the past decade. In 2000, the Ministry of Public Health did not have a mental health unit or a mental health policy or plan. The entire country had just one psychiatrist, and altogether lacked psychiatric nurses and psychiatric social workers.

A pilot project started in 2000 to provide mental health services in one area of the country. The project was spearheaded by a NGO and started under fragile financial conditions: the majority of its funding came from a single international donor. In addition, the project challenged traditional ways of understanding and providing mental health care in the country. Health professionals were not generally familiar with community-based mental health care. A new cadre was introduced – psychosocial workers – and more specialized services were provided through monthly mental health clinics in provincial hospitals. As a result of these services, more than 27 000 people were helped from 2000 to 2008 [13].

The transition of mental health services from the NGO to the government, which occurred between 2007 and 2010, presented formidable challenges. The government was faced with funding problems, and political instability hampered decision-making. Nonetheless, a national mental health strategy and policy were adopted, and the government is now supplying essential psychiatric medications through its national drug distribution centre.

### Indonesia (Aceh)

Aceh suffered from decades of conflict before being struck by a tsunami in 2004. Prior to this disaster, mental health care was available only through the province’s sole mental hospital, located in the capital city of Banda Aceh. This mental hospital was institutional in nature, with one part-time and two full-time psychiatrists, 220 beds, and an occupancy rate usually exceeding 100%. Meanwhile, primary and secondary health centres had non-existent capacity to deliver mental health services.

In the aftermath of the tsunami, hundreds of NGOs sent teams to provide various forms of mental health and psychosocial support. Despite the chaos, the government and selected actors saw an opportunity to start building a mental health system that would serve the long-term needs of the population beyond the recovery phase [23]. Indonesia’s Ministry of Health and WHO provided leadership and specific recommendations on building a comprehensive mental health system. Diverse stakeholders came together and agreed to work together to develop mental health services in primary health care – using in part the work already developed and implemented by the International Medical Corps in one district – while improving the quality of care in the sole existing mental hospital, and later providing care in certain district general hospitals. Community mental health nurses provided basic care and were supported by medical officers, who received a refresher course on primary care psychiatry.

Today, Aceh has in most districts a public mental health infrastructure with primary health services supported by secondary care at district general hospitals. The majority of Aceh’s districts have specific mental health budgets, compared with none a decade ago. Aceh is viewed as a role model for mental health for other provinces in Indonesia, and serves as a prime example of how the influx of resources following an emergency can be used to strengthen the mental health system.

### Iraq

Prior to the invasion in 2003, mental health care in Iraq was provided through mental hospitals and in university-affiliated settings. In 2004, a National Mental Health Council was formed and led efforts to rebuild the country’s mental health system. One of their most important recommendations was to create a mental health system that was radically different from the country’s previous one. This included transforming the institutional, biomedically-based model of mental health care into an integrated, community-based approach.

Significant progress was made to create a more comprehensive mental health system, including the establishment of community mental health centres in all governorates, integration of mental health services within primary health care, and reform of psychiatric institutions. Table 1 displays advancements made during this period.

**Table 1:** **Comparison of mental health services in Iraq prior to 2003 versus 2011**

**Mental health service level**	**Prior to 2003**	**2011**
Long-stay facilities and specialist services	Two institutional-style mental hospitals in Baghdad.	Baghdad mental hospitals still exist; undergoing reform.
Psychiatric services in general hospitals	Outpatient services only, and limited to large hospitals in the centres of the governorates.	25 new mental health units, offering mix of inpatient and outpatient services.
		New inpatient beds for children and adolescents in paediatric hospital.
Community mental health services	Public outpatient services in major general and university hospitals.	34 new outpatient-only units, including:
	Private clinics in main cities for those who could afford them.	• 4 for children and adolescents;
		• 1 for maternal mental health;
		• 1 for geriatric mental health;
		• 8 for trauma counselling;
		• 1 for substance abuse rehabilitation and treatment.
Primary care services for mental health	Minimal.	Integration of mental services within primary health care started throughout Iraq.

Ongoing violence and lack of security in many parts of Iraq have undermined the ability to implement service reform in certain geographic areas and have challenged the continuity of mental health reform. The lack of continuous financial support for mental health reform has been another issue. Nonetheless, several donors have stepped forward to support mental health development activities and projects.

### Jordan

Waves of displaced Iraqis flowed into Jordan after 2003; this influx drew the attention and support from international donors and aid agencies. Basic health care, psychosocial support, education, and recreational activities were among the services provided. Although well-intentioned, the long-term sustainability of many of these programmes was of concern, as they created a system that was parallel to – but not integrated within – the Jordanian public health system.

During that time, Jordan’s mental health system was highly centralized and mainly based on psychiatric hospitals. Some outpatient services were available, but predominantly restricted to large cities and overall insufficient.

A small portion of incoming donor funds was used to establish in 2008 to 2009 three multidisciplinary, community-based mental health centres for Iraqi and Jordanian people. From 2009 to 2011, 3550 people received comprehensive treatment in these centres. Word spread and the centres started receiving referrals from NGOs, primary health care centres (PHCCs), and the community. This is turn attracted the attention of the media, international donors, and government officials; momentum was built for broader change across the country. A National Steering Committee for Mental Health was formed, and a national mental health policy and plan were launched in 2011. Since that time, around 200 primary health care workers have been trained to deliver mental health services in PHCCs using the WHO Mental Health Gap Action Programme Intervention Guide (mhGAP-IG). Courses are now being delivered by local trainers.

One challenge to mental health reform in Jordan – as in other countries – was initial reluctance among a number of mental health specialists. The reform posed a challenge to the prevailing biomedical treatment approach, as it promoted comprehensive, biopsychosocial treatment and introduced and emphasized the role of multidisciplinary teams. This challenge was addressed to a large extent through several means: involving all psychiatrists in the reform process; relying on supportive “champions” to serve as change agents within their fields; harnessing the motivation and determination of other mental health professionals to support reform; and benefiting from strong support at the highest political level.

This case illustrates that mental health reform need not begin with a national policy or plan. In Jordan, a small-scale pilot project was the first step leading to broader reform.

### Kosovo

Kosovo suffered from substantial violence and instability that came to a head in 1999. Rapid political change generated an opportunity to reform Kosovo’s mental health system, which until that time was biologically focused and hospital-based. While WHO clarified the kind of approach it advocated and the support it was ready to provide, Kosovar neuropsychiatrists created a Mental Health Task Force that guided efforts. Following extensive consultations, a Mental Health Strategic Plan was finalized in 2000 and approved in 2001. Numerous organizations and government donors contributed to the reform in different ways. The Mental Health Strategic Plan was the roadmap through which all actions could be coordinated.

Despite a common framework for action, resistance from others had to be overcome on several fronts. For example, some thought asylums were necessary; others were interested only in post-traumatic stress disorders or the promotion of psychosocial well-being; yet others believed that the Kosovar community was not prepared to sustain a community-based mental health system. Despite these challenges, the process moved forward and ten years later it is still vibrant, due largely to the focus on sustainability and the commitment of local professionals.

Today, each of Kosovo’s seven regions offers a range of community-based mental health services (see Table 2), and Kosovo’s asylum has been transformed. This case underscores the importance of engaging local professionals and shows how a detailed mental health strategic plan can function as a roadmap through which diverse stakeholders and projects can be coordinated.

**Table 2:** **Summary of facility changes in Kosovo’s public mental health sector, 2000 to 2010**

**Type of facility**	**2000**	**2005**	**2010**
Community-based mental health centres for adults	0	7	9
Inpatient wards in general hospitals for adults	6	6	6
Residential facilities for adults	0	4	8
Community-based mental health centres for children and adolescents	0	1	1
Primary health care units for children and adolescents	0	2	2
Residential facilities for children and adolescents	0	2	2
Asylums	1	0	0

### Occupied Palestinian territory

The Palestinian population’s mental health needs are considerable and are exacerbated by continued conflict and violence; high population density with a significant proportion of the population living as refugees; unemployment, social deprivation, and poverty; and ongoing loss, traumatic events, and human rights violations.

In 2000, most mental health resources were concentrated in tertiary psychiatric hospitals, despite some community mental health clinics in the West Bank and Gaza Strip. Primary health workers had little or no training in mental health care.

Since that time, significant improvements have been made. Supported by government plans, the system is moving towards community-based mental health care. Ten new centres have opened across the area, funded by a range of donors. Psychiatric hospitals are being reformed, and training has been conducted in primary care clinics and community mental health centres for 580 health workers.

Mental health services have struggled with intermittent conflict and unrest, as well as with the physical and political separation between the West Bank and Gaza Strip. Additional support is needed to consolidate progress made, in particular to continue the training and supervision of general practitioners and nurses working in primary care, to further implement the primary care approach to service delivery. Despite these challenges, flexibility with implementation has enabled the continued strengthening of community-based mental health services across the territory.

### Somalia

This country has been in a complex emergency for more than 20 years. Most people with mental disorders do not receive any sort of modern mental health care and many experience significant human right violations.

Due to the ongoing crisis and weak governance structures, full reform of the mental health system has not been possible. Nonetheless, various projects – including situation analyses, a mental health strategy, a chain-free initiative, and health worker training – helped improve mental health care. More than 1700 people with mental disorders had their chains removed between 2007 and 2010; 55 health workers were trained in mental health care; and two health worker participants are now mental health coordinators/focal points for parts of the country. Participants are also managing three new mental health facilities. And importantly for the future, mental health is now included in the medical school curricula of two universities.

The lack of an effective central governance structure is among the greatest challenges to further reform; more specifically, lack of funds and infrastructure has inhibited larger-scale improvement. This case demonstrates nonetheless that important improvements in mental health services can happen in contexts where full-scale national mental health reform is not possible.

### Sri Lanka

Most mental health services in Sri Lanka were provided through tertiary-level hospitals in major cities prior to 2004. This meant that most people with mental health needs failed to receive any sort of treatment.

Following the 2004 tsunami, Sri Lanka’s head of state recognized the need to address the acute psychological distress of survivors. Fuelled by international media interest and rapid resolution of other health issues, mental health became a prominent part of the political agenda. A new national mental health policy was adopted shortly after the disaster and guided reform. It emphasized comprehensive, decentralized, and community-based mental health care, and called for several new cadres of mental health workers.

The lack of trained mental health professionals was one of the greatest challenges in implementing Sri Lanka’s mental health reform. To address this issue, partners worked together to establish a one-year diploma course in psychiatry and to train Medical Officers of Mental Health, Medical Officers of Psychiatry, community mental health nurses, and community support officers.

Although mental health has lost prominence within the political agenda compared with the year following the tsunami, national and international investments in mental health have continued. This has enabled Sri Lanka to continue to build on the early momentum.

**Figure 1:**
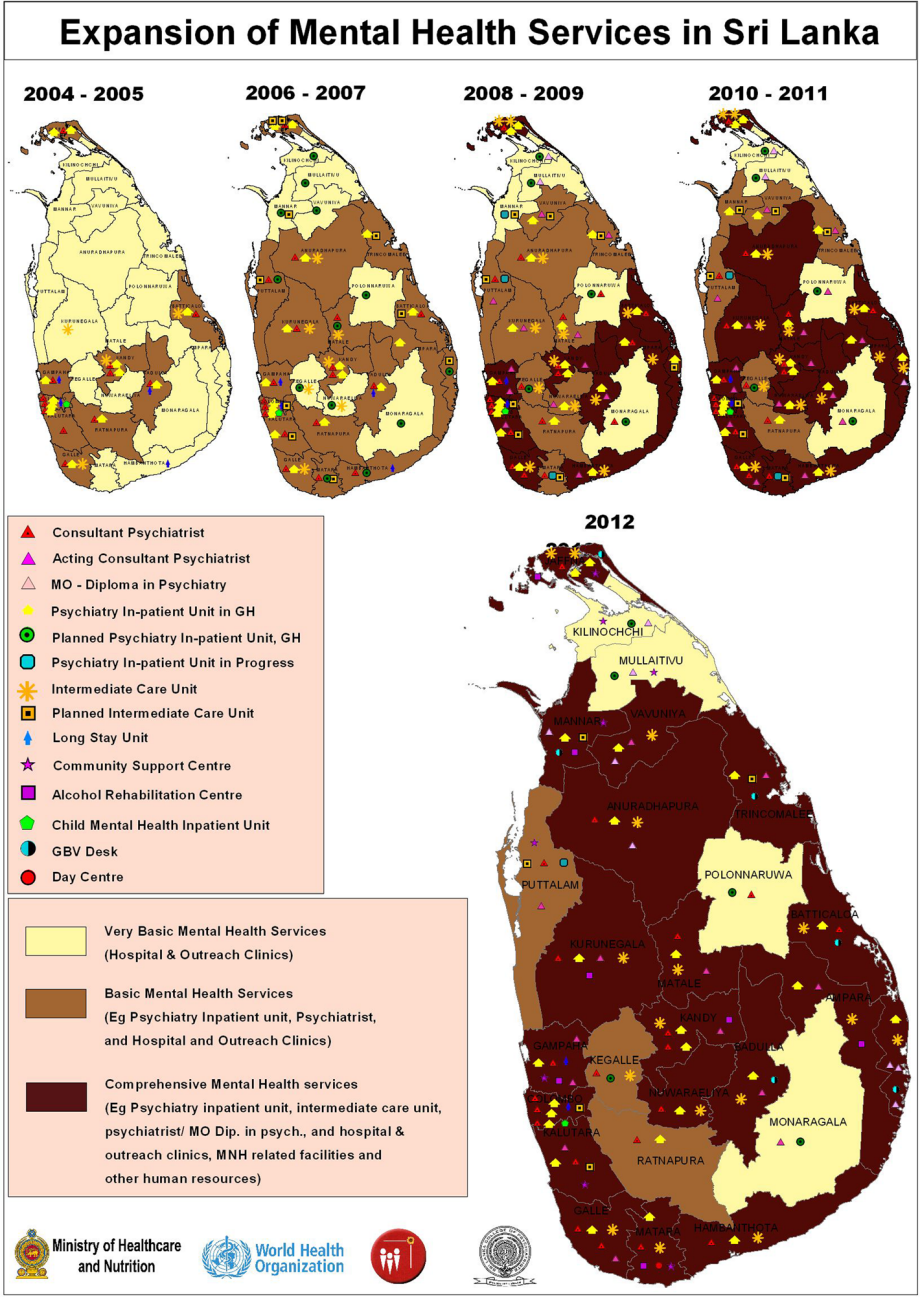
**Expansion of mental health services in Sri Lanka, 2004 to 2012.**

Reforms continue to expand in coverage and now extend beyond the tsunami-affected zones to most parts of the country (see Figure 1). As of 2011, 20 out of 26 health districts (77%) had functioning acute inpatient units within general hospital settings, compared with 10 out of 26 (38%) before the tsunami. In addition, the country had 16 fully functional intermediate stay rehabilitation units, compared with five units in 2004.

### Timor-Leste

Prior to achieving independence in 2002, mental health services were wholly absent in Timor-Leste. Although this meant that Timorese people did not have access to mental health treatment, it created an opportunity to establish an evidence-based mental health service system.

A consortium of agencies from outside the country led initial efforts. Faced with a blank slate and a limited budget, they focused on the training and support of health workers to provide effective community-based mental health services. They elected to defer the establishment of inpatient facilities, and to be organizationally independent of primary care facilities, which had been severely damaged and were struggling to provide even the most rudimentary care. Priority attention was given to those with severe mental disorders.

A multidisciplinary team of mental health professionals from Australia and other countries provided extensive input into training and supervision of mental health workers from 2000 to 2005. In addition to classroom-based training, psychiatrists and other mental health professionals spent extensive time in the field working side-by-side with Timorese colleagues.

With the termination of international donor funding, it was no longer possible to maintain the systematic programme of international support. The Ministry of Health subsequently assumed these functions and made further progress in developing services.

A new National Mental Health Strategy (2010–2020) is now part of the Ministry of Health’s overall long-term strategic plan. Mental health services are community-based and organized across different levels of the health system. More than 4000 people received mental health services in 2011 and more mental health workers continue to be trained.

## Discussion and evaluation

The 10 cases described above show that despite challenges, it is possible to make gains in building and strengthening sustainable mental health services during and following emergencies.

Although they differed in terms of their underlying socio-political contexts and hazards causing the emergency, common lessons learnt could be identified. These are detailed below. For this article, the 10 lessons learnt were assessed for their applicability by the co-author with the most direct experience in each different emergency; these results are displayed in Table 3.

**Table 3:** **The applicability of 10 lessons learnt across 10 diverse emergency-affected areas**

		**Afghanistan**	**Burundi**	**Indonesia (Aceh)**	**Iraq**	**Jordan**	**Kosovo**	**Occupied Palestinian territory**	**Somalia**	**Sri Lanka**	**Timor-Leste**
1.	Mental health reform was supported through planning for long-term sustainability from the outset	✓	-	✓	✓	✓	✓	✓	-	✓	✓
2.	The broad mental health needs of the emergency-affected population were addressed	✓	✓	✓	✓	✓	✓	✓	-	✓	✓
3.	The government’s central role was respected	✓	✓	✓	✓	✓	✓^1^	✓	-	✓	✓
4.	National professionals played a key role	✓	✓	✓	✓	✓	✓	✓	✓	✓	-
5.	Coordination across agencies was crucial	✓	-	✓	✓	-	✓	✓	✓	✓	-
6.	Mental health reform involved review and revision of national policies and plans	✓	-	-	✓	✓	✓	✓	✓	✓	✓
7.	The mental health system was considered and strengthened as a whole	✓	-	✓	✓	✓	✓	-	-	✓	✓
8.	Health workers were reorganized and trained	✓	✓	✓	✓	✓	✓	✓	✓	✓	✓
9.	Demonstration projects offered proof of concept and attracted further support and funds for mental health reform	✓	✓	✓	✓	✓	✓	✓	✓	✓	✓
10.	Advocacy helped maintain momentum for change	✓	✓	✓	✓	✓	✓	✓	✓	✓	-

Table 3 note:^1^ Kosovo was initially a United Nations (UN) protectorate, during which all agreements were made with local professionals and the UN. Later, a consistent administrative organization was created within the Ministry of Health and the Regional Health Authorities.

1. **Mental health reform was supported through planning for long-term sustainability from the outset.**
  Most cases demonstrated how early commitment to mental health reform played a role in creating long-term sustainability. Although emergency funding is often short term, some key donors were prepared to facilitate the transition to funding for longer-term mental health reform through repeated contracts. In other cases, the government assumed financial responsibility for continuing mental health care in the longer term.
2. **The broad mental health needs of the emergency-affected population were addressed.**
  Reforms generally covered a wide range of mental health problems, as opposed to only one disorder (e.g. post-traumatic stress disorder). A focus on broad mental health needs was relevant, because adversity is a risk factor for most mental disorders, including psychosis, and because most affected areas had limited services for those with pre-existing mental disorders.
3. **The government’s central role was respected.**
  The preponderance of cases described working from within the government to enact reform. This included in some cases strengthening of governance structures through seconding professional staff and temporarily assigning specific functions to nongovernmental organizations under government oversight.
4. **National professionals played a key role.**
  In most cases, local professionals were powerful champions in promoting and shaping mental health reform. Helpful international experts and agencies involved themselves in mental health reform only to the extent that they were invited to do so.
5. **Coordination across agencies was crucial.**
  Coordination of actors interested in long-term mental health development was typically crucial when working towards mental health reform. Proactive efforts in several cases facilitated consensus among diverse multisectoral partners, who then worked from an agreed framework. This type of coordination increased the likelihood for partners to complement – as opposed to duplicate – one another by taking different areas of responsibility.
6. **Mental health reform involved review and revision of national policies and plans.**
  Most cases described an overall integrated public health approach that involved mental health policy reform. And in the context of disaster, where political will for mental health care was high, the reform process was typically accelerated.
7. **The mental health system was considered and strengthened as a whole.**
  Many cases described processes that reviewed and assessed the mental health system as a whole, from community- to tertiary care-levels. Doing so provided an understanding of the overall system and how it was affected by the emergency. This then formed the basis for planning system-wide strengthening of services.
8. **Health workers were reorganized and trained.**
  Opportunities arose in all post-emergency contexts to reorganize, train, and provide ongoing supervision to health workers so that they were better equipped to manage mental health problems. This involved training and supervising health staff, as well as creating new health worker cadres to fill service gaps. Health workers recruited from local communities helped ensure long-term tenure and culturally appropriate, acceptable interventions.
9. **Demonstration projects offered proof of concept and attracted further support and funds for mental health reform.**
  Demonstration projects provided proof of concept in all cases. They also helped ensure momentum for longer-term funding. The latter was particularly true when the demonstration projects were explicitly linked to discussions and plans on broader mental health reform.
10. ** Advocacy helped maintain momentum for change.**
  In almost all cases, key individuals or groups played crucial roles in advocating for broader mental health reform. They helped maintain momentum for change after the acute emergency. Advocacy was most successful when diverse groups of people were not only informed about the issues, but also asked to become part of the solution.

Although these cases occurred between 2000 and 2010, their lessons learnt are arguably just as relevant today. Major emergencies continue to occur, and affected areas face similar challenges to those encountered in the cases described above.

Nonetheless, some limitations should be noted. These cases are not the result of an exhaustive review: successful progress with mental health service development during or following emergencies has been reported in other countries [24-30]. Second, the lessons learnt were developed and validated using an informal process of consultation among co-authors. We cannot rule out the possibility that a different set of cases and/or co-authors might have emphasized somewhat divergent lessons learnt. Third, caution must be used in generalizing results. For example, the WHO regions of the Americas and the Western Pacific were not represented among the cases. Moreover, these cases were chosen in part *because* they were successful in building better mental health care. All-too-often, mental health and psychosocial assistance following emergencies is short term only, and cases of such situations were not analysed here.

Notwithstanding these limitations, the 10 cases profiled in this article show that it is possible to make long-term improvements to mental health care following emergencies. While interest in mental health tends to be particularly large after natural disasters, this collection of cases shows that substantial opportunities for mental health reform also exist after conflict.

## Conclusion

Collectively, these 10 cases support the notion that emergency situations, despite the cascade of human suffering they create, are opportunities to catalyse mental health service development. This was demonstrated even in areas with extremely weak mental health systems and/or complex humanitarian crises.

Global progress will happen more quickly if, in every emergency, strategic efforts are made to convert short-term interest in the mental health needs of survivors into momentum for mental health reform. Information and tools [5,8,31-33] are available to help guide action.

Although reform requires relatively modest financial investment, steadfastness in long-term goal is crucial amidst the sudden surge of aid agencies into affected areas, most of which are concerned with short-term relief only. Two types of investments are needed: one-time start-up costs; and continuous service delivery costs. It is the one-time start-up costs that can be financed by short-term external funding provided in emergencies.

These are opportunities not to be missed. Taking action would benefit not only people’s mental health, but likely also the functioning and stability of societies recovering from emergencies.

## Abbreviations

NGO: Nongovernmental organization
PCCCs: Primary health care centres
WHO: World Health Organization
